# 4See: A Flexible Browser to Explore 4C Data

**DOI:** 10.3389/fgene.2019.01372

**Published:** 2020-01-21

**Authors:** Yousra Ben Zouari, Angeliki Platania, Anne M. Molitor, Tom Sexton

**Affiliations:** ^1^ Institute of Genetics and Molecular and Cellular Biology (IGBMC), Illkirch, France; ^2^ CNRS UMR7104, Illkirch, France; ^3^ INSERM U1258, Illkirch, France; ^4^ University of Strasbourg, Illkirch, France

**Keywords:** 4C, epigenomics, browser, chromatin loops, quantile normalization, biological replicates

## Abstract

It is established that transcription of many metazoan genes is regulated by distal regulatory sequences beyond the promoter. Enhancers have been identified at up to megabase distances from their regulated genes, and/or proximal to or within the introns of unregulated genes. The unambiguous identification of the target genes of newly identified regulatory elements can thus be challenging. Well-studied enhancers have been found to come into direct physical proximity with regulated genes, presumably by the formation of chromatin loops. Chromosome conformation capture (3C) derivatives that assess the frequency of proximity between different genetic elements is thus a popular method for exploring gene regulation by distal regulatory elements. For studies of chromatin loops and promoter-enhancer communication, 4C (circular chromosome conformation capture) is one of the methods of choice, optimizing cost (required sequencing depth), throughput, and resolution. For ease of visual inspection of 4C data we present 4See, a versatile and user-friendly browser. 4See allows 4C profiles from the same bait to be flexibly plotted together, allowing biological replicates to either be compared, or pooled for comparisons between different cell types or experimental conditions. 4C profiles can be integrated with gene tracks, linear epigenomic profiles, and annotated regions of interest, such as called significant interactions, allowing rapid data exploration with limited computational resources or bioinformatics expertise.

## Introduction

Since early transgenic studies it has been clear that promoter sequences are insufficient to regulate the spatiotemporal expression patterns of many developmental genes. “Remote control” is additionally conferred by distal activating sequences, termed enhancers, which have been intensively studied over the last years (Schoenfelder and Fraser, 2019). Genome-wide profiling of histone modifications and protein binding sites by ChIP-seq have uncovered a general chromatin signature of enhancer regions: DNase-hypersensitive, bound by the transcriptional coactivator p300, and marked by the monomethylation of lysine-4 of histone H3 (H3K4me1) (Heintzman et al., 2009). Follow-on studies refined these findings further by identifying chromatin features that were characteristic of different enhancer properties. For example, the strongest-acting enhancers are also accompanied by acetylation of lysine-27 of histone H3 (H3K27ac) (Creyghton et al., 2010; Rada-Iglesias et al., 2011) and/or acetylation on globular histone domains (Pradeepa et al., 2016), recruit RNA polymerase II, and general transcriptional machinery (Koch et al., 2011), and are even transcriptionally active, producing non-coding RNA (eRNAs) (Kim et al., 2010). Enhancers lacking these extra features, and sometimes even encompassing repressive marks, such as H3K27 trimethylation (H3K27me3), are proposed to be “poised” enhancers, which may become activated at later developmental stages. Interestingly, the chromatin states at enhancer sequences vary much more across cell types than those of gene promoters (Roadmap Epigenomics et al., 2015), suggesting that much of the regulatory potential is epigenetically carried by enhancers. However despite advances in identifying enhancers genome-wide, both through epigenomic profiling and high-throughput reporter assays (Arnold et al., 2013; Roadmap Epigenomics et al., 2015), unambiguous identification of their target genes is still a major challenge. Important developmental enhancers have been found at megabase distances from target genes, and/or within the introns of unregulated genes (Lettice et al., 2003; Amano et al., 2009; Herranz et al., 2014); previous studies estimate that up to ~90% of enhancers may indeed skip the closest genes on the linear chromosome fiber (Sanyal et al., 2012; Schoenfelder et al., 2015).

Since the advent of the chromosome conformation capture method (3C) (Dekker et al., 2002) and its variants to measure relative spatial proximity of pairwise genomic regions, many enhancers have been found to physically interact with their target genes, often with “looping out” of the intervening chromatin (Palstra et al., 2003); the resultant “active chromatin hub” has been proposed to provide the permissive regulatory environment for transcription initiation, although the exact mechanism remains unclear. In many studied cases, looping is concomitant with transcriptional induction, whereas in others, the loop is pre-formed to poise the gene for subsequent activation (Schoenfelder and Fraser, 2019). Recent reports using microscopy methods have also been made of enhancers and promoters being well separated on gene activation (Alexander et al., 2019; Benabdallah et al., 2019), although enhancer-promoter interactions were previously reported in the studied loci, raising questions as to whether interactions may completely precede transcription and/or be very transient events. In any case, physical proximity measured by 3C-based methods is becoming a popular means of ascribing target genes to otherwise cryptic distal regulatory elements, or of identifying novel candidate regulatory regions of specific genes of interest. For example, intergenic sequence variants associated with diseases have been better characterized once their target genes were identified by 3C-based approaches (Herranz et al., 2014; Schoenfelder and Fraser, 2019).

**Figure 1:**
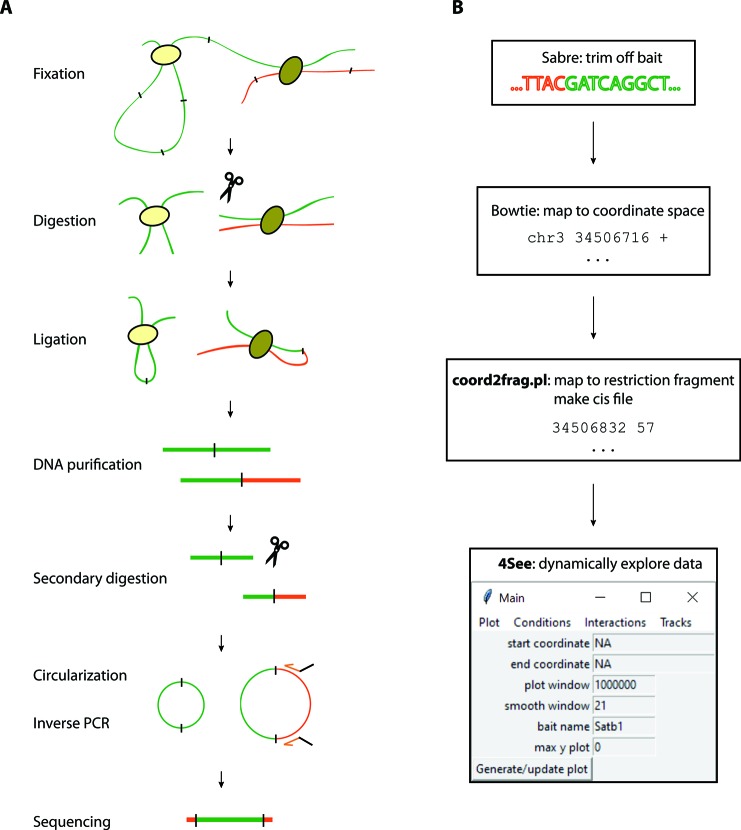
Overview of the 4C method and analysis. **(A)** The 4C method entails chromatin fixation, restriction digestion and re-ligation to generate hybrid sequences between fragments that were physically proximal during fixation. The DNA is purified, digested with a secondary restriction enzyme and re-ligated under dilute conditions to generate DNA circles. Chimeric products linked to a specific bait fragment of interest (orange) are amplified by inverse PCR with bait-specific primers (orange arrows) flanked by Illumina sequencing adapters (black overhangs). The PCR products are then directly loaded onto Illumina flow-cells for high-throughput sequencing. **(B)** Pre-processing steps before 4See; tools denoted in bold accompany this manuscript. The fastq sequences are first trimmed to remove bait restriction fragment sequence (orange), leaving just the prey DNA sequence (green) for mapping to the reference genome with Bowtie. The mapped genomic coordinates are converted to restriction fragment space by a custom perl script, which counts the total number of reads mapping to each fragment on the same chromosome as the bait. This “cis” file can then be directly input into 4See.

With the advent of next-generation sequencing, several higher throughput variants of 3C have been developed to obtain genome-wide chromatin interaction maps. Hi-C is an “all-to-all” method, systematically assessing all pairwise chromatin contacts (Lieberman-Aiden et al., 2009). However, due to the great complexity of the sequenced material, calling specific looping interactions requires prohibitively expensive sequencing depth (Rao et al., 2014; Bonev et al., 2017), and Hi-C loop calling algorithms have been demonstrated to not be very robust (Forcato et al., 2017). A recent modification, Capture Hi-C, incorporates capture with a pool of thousands of oligonucleotides, allowing the complexity of sequenced Hi-C material to be reduced sufficiently to assess the chromatin looping interactions with all promoters (Hughes et al., 2014; Sahlen et al., 2015; Schoenfelder et al., 2015). However, capture libraries can be expensive, and their design still represents a trade-off between coverage of assessed promoters and resolution of the identified loops. For the highest resolution profiling of smaller numbers of candidate regions, the method of choice is the “one-to-all” 4C (circular chromosome conformation capture), which assesses all the chromatin interactions with one specific bait of interest (Simonis et al., 2006; van de Werken et al., 2012) (**Figure 1A**). In brief, nuclei are fixed in their native topologies with formaldehyde, digested with a restriction enzyme and re-ligated, as for 3C, such that chimeric DNA sequences are generated between restriction fragments which may be unlinked on the linear chromosome fiber but are physically proximal at the time of fixation. The purified DNA is then circularized by digestion with a secondary restriction enzyme and re-ligation under dilute conditions, allowing an inverse PCR strategy to amplify all the chimeric DNA linked to a specific bait restriction fragment of interest. The much reduced complexity of a 4C library, compared to that of Hi-C, means that promoter interactomes can be reliably profiled with just a few million sequence reads, and ~20 baits can readily be multiplexed into a sequencing run, making it a much more cost-effective method (van de Werken et al., 2012). The major limitations of 4C are the relatively small throughput in baits that can be assessed at a time, and that the direct sequencing of PCR products confounds results with large numbers of PCR duplicates that cannot be distinguished from counts of true 3C ligation events. However, *in silico* approaches can minimize the impact of PCR duplicates (de Wit et al., 2015), and “unique molecular identifier” variants of 4C have also been developed (Schwartzman et al., 2016).

**Figure 2:**
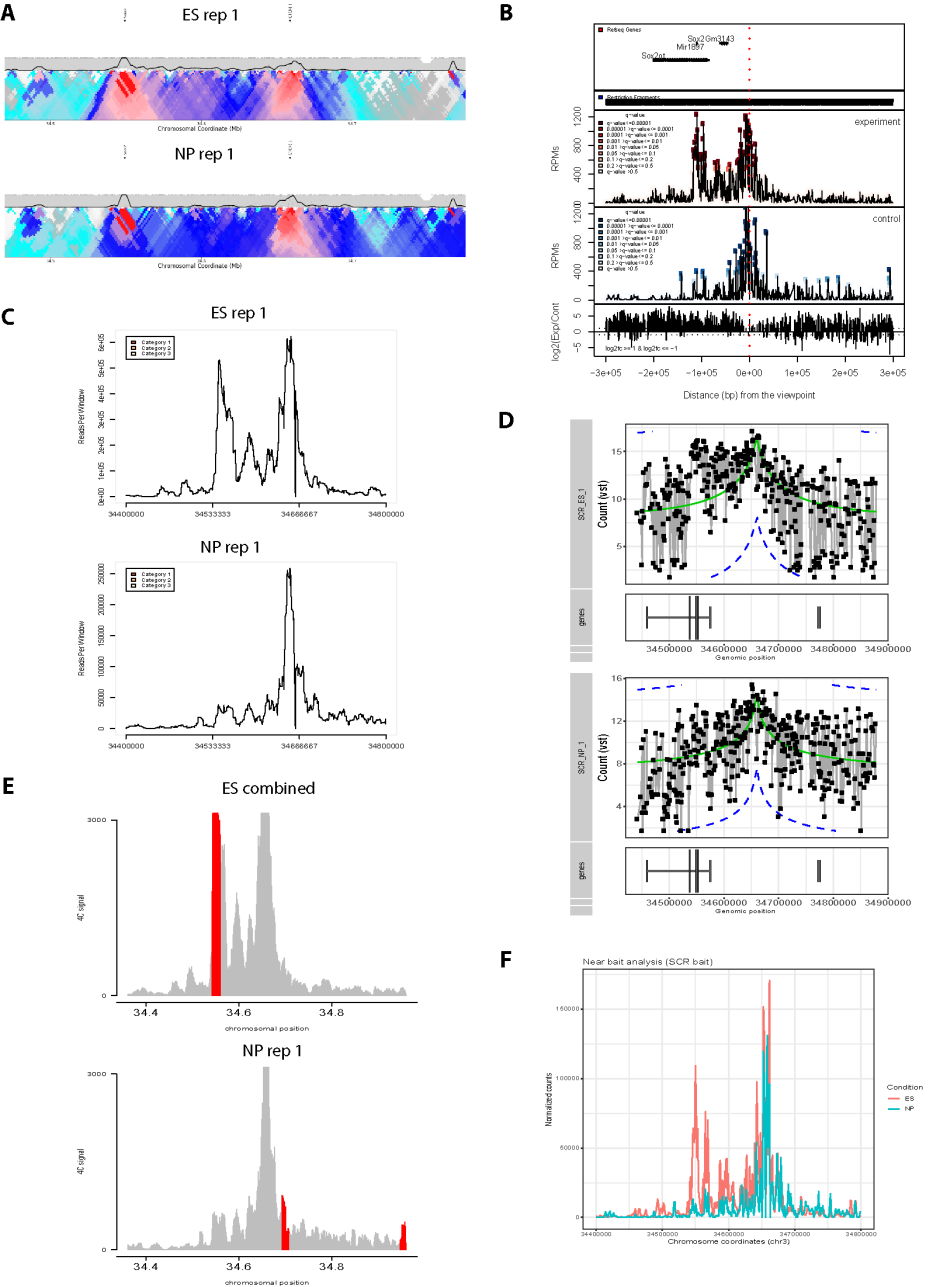
Overview of graphing options from existing 4C analysis methods. All methods have been applied to two ES replicates and one NPC 4C data set for the *Sox2* SCR bait (see also **Figure 3**). **(A)** 4Cseqpipe (van de Werken et al., 2012) results shown independently for one ES replicate and the NPC data set. Running median scores are plotted as a line graph (5 kb resolution), with domainograms plotted underneath as a heat map for median scores at steadily increasing window sizes. Positions of the CTCF site within the SCR and the *Sox2* gene are indicated by arrows. Note that the independent normalization means that the SCR-*Sox2* interaction differences between the two cell types is not evident, compared to other methods. **(B)** r3Cseq (Thongjuea et al., 2013) results for the combined three data sets, showing panels, from top to bottom: positions of Refseq genes; restriction fragment coverage; averaged profile for the “experiment” (ES) condition, with called interactions at different confidence levels highlighted; profile for the “control” (NPC) condition, with called interactions at different confidence levels highlighted; plot of the log2-ratio of experiment vs control 4C signal. The position of the SCR bait is indicated by a red dashed line. Note that r3Cseq appears to call a very large number of interactions. **(C)** fourSig (Williams et al., 2014) results shown independently for one ES and one NPC replicate, showing smoothed 4C plots (21-fragment windows). Called interactions (“Categories” 1, 2 or 3, for different confidence levels) would be highlighted on the plot, but none were called by fourSig. **(D)** FourCSeq (Klein et al., 2015) results shown independently for one ES and one NPC replicate, showing normalized and processed 4C signal plots (gray line graphs and black points), alongside the positions of known genes. The green line indicates the centralized 4C value, and the dashed blue lines indicate the threshold values for a z-score difference > 2. Significant interactions would be highlighted, but were not detected by FourCSeq. Note that differences between the two cell types is not so evident, compared to most other methods. **(E)** peakC (Geeven et al., 2018) results shown for the combined analysis of the two ES replicates, independently of the NP replicate, giving smoothed 4C plots (21-fragment windows) as histograms. The red regions indicate called interactions. **(F)** 4C-ker (Raviram et al., 2016) results shown for the combined analysis, with line plots of combined ES (red) and NPC (blue) 4C signal. Note that many interactions were called by 4C-ker within the plotted window for both ES and NP, but that the documentation did not provide a means to plot them alongside the shown line plots.

Due to the growing popularity of 4C experiments, several algorithms have been developed to call significant interactions (van de Werken et al., 2012; Thongjuea et al., 2013; Williams et al., 2014; Klein et al., 2015; Raviram et al., 2016; Geeven et al., 2018); recent benchmarking shows that all methods work well on simulated data, but no single method is optimum for all experimental setups (Walter et al., 2019). However, whereas most of these methods has an in-built tool to plot the static results after data processing, a simple, flexible browser allowing a user to rapidly visualize their 4C results is currently lacking (see **Figure 2** and summary of the different plotting options currently available in **Table 1**). Moreover, while some methods allowed raw and/or smoothed 4C data to be exported as files that can be opened and visualized alongside epigenomic profiles on genome browsers, they offered no flexibility in plotting the epigenomic profiles directly alongside the 4C plot while different smoothing or peak calling parameters are being trialed. We recently developed ChiCMaxima, a suite of tools to analyze Capture Hi-C data, which includes a GUI (graphical user interface) to flexibly visualize data sets alongside gene annotations and epigenomic profiles (Ben Zouari et al., 2019). Here we report 4See, the adaptation of ChiCMaxima tools for the user-friendly integrative exploration of 4C data sets. 4See provides flexibility to compare different replicates side by side, or to average them together when comparing experimental conditions, and to visualize 4C profiles at different smoothing window sizes, necessary to identify putative interactions at different distances from the bait, without the need to reload or re-process the initial data. 4See utilizes quantile normalization to allow different plotted profiles to be fairly compared during the visualization. 4See also allows 4C profiles to be easily plotted alongside gene annotations and linear epigenomic tracks, as well as for specific regions (e.g. interactions called by other algorithms) to be highlighted. We anticipate that 4See will be a useful tool to the community for quick and easy exploration of 4C data, particularly when used in conjunction with existing interaction calling tools.

**Table 1:** Comparison of graphing options from existing 4C analysis methods.

Method	GUI	Preprocessing	Handles conditions/replicates	Plotting options	Annotations
4Cseqpipe	No	Several custom scripts to convert fastq files to multiple formats and intermediate files. These failed for test data and intermediate files had to be made manually.	No	Can alter trendline resolution and plotting window (coordinate space); domainogram parameters fixed	Limited: manually curated bed file gives arrows on plot
r3Cseq	No	Processes bam files directly	Yes, but restricted to pairwise comparison of “experiment” and “control” conditions	Can only alter plotting window	RefSeq genes
fourSig	No	Custom script converts bam to input format	No	Can only alter plotting window	None
FourCSeq	No	Need to set up metadata table in R, which points to processed bam files	Handled in one combined object, but default is to plot each data set individually, and documentation does not say how to do otherwise.	Can only alter plotting window	Positions of genes from transcriptome (unlabeled)
4C-ker	No	Requires bed file of restriction fragments and bedgraph of 4C coverage per observed fragment. Custom scripts to generate from sam files failed and input files had to be made manually.	Yes	In principle, many settings can be changed in the R command prompt (ggplot2 call settings), but is not documented or user-friendly	None
peakC	No	Essentially the same as this manuscript, but utility scripts not provided	Handles replicates but not different conditions	Can only alter plotting window	None

## Materials and Methods

### Pre-Processing

#### 4C

J1 mouse embryonic stem (ES) cells were grown on gamma-irradiated mouse embryonic fibroblast cells under standard conditions (4.5 g/L glucose-DMEN, 15% FCS, 0.1 mM non-essential amino acids, 0.1 mM beta-mercaptoethanol, 1 mM glutamine, 500 U/mL LIF, gentamicin), then passaged onto feeder-free 0.2% gelatin-coated plates for at least two passages to remove feeder cells. For *in vitro* differentiation to neural precursor cells (NPCs), F1 ES cells were cultured in the same medium supplemented with 1 µM PD03259010 and 3 µM CHIR99021 (“2i” conditions) and without feeders. The cells were then cultured for six days with medium without LIF or 2i and with 10% FCS, and with 5 µM retinoic acid for the final four days, to generate embryoid bodies (Bibel et al., 2007). J1/F1 ES or differentiated cells were detached with trypsin, then washed by centrifugation in PBS before fixation. Mouse CD4^+^ CD8^+^ double-positive (DP) thymocytes were obtained from 4 week old mouse thymus by FACS with anti-CD4-PE and anti-CD8a-FITC antibodies (eBioScience). Both cell preparations were fixed with 2% formaldehyde in mES culture medium for 10 min at 23°C. The fixation was quenched with cold glycine at a final concentration of 125 mM, then cells were washed with PBS and permeabilized on ice for 1 h with 10 mM Tris-HCl, pH 8, 100 mM NaCl, 0.1% NP-40, and protease inhibitors. Nuclei were resuspended in *Dpn*II restriction buffer at 10 million nuclei/mL concentration, and 5 million nuclei aliquots were further permeabilized by treatment for either 1 h with 0.4% SDS at 37°C (ES cells), or for 20 min with 0.7% SDS at 65°C, then for 40 min at 37°C (DP cells). The SDS was then neutralized by incubating for a further 1 h with either 2.6% (ES) or 3.3% (DP) Triton-X100 at 37°C. Nuclei were digested overnight with 1000 U *Dpn*II at 37°C, then washed twice by centrifuging and resuspending in T4 DNA ligase buffer. *In situ* ligation was performed in 400 μL T4 DNA ligase buffer with 20,000 U T4 DNA ligase overnight at 16°C. DNA was purified by reverse cross-linking with an overnight incubation at 65°C with proteinase K, followed by RNase A digestion, phenol/chloroform extraction, and isopropanol precipitation. The DNA was digested with 5 U/μg *Csp*6I at 37°C overnight, then re-purified by phenol/chloroform extraction and isopropanol precipitation. The DNA was then circularized by ligation with 200 U/μg T4 DNA ligase under dilute conditions (5 ng/μL DNA), and purified by phenol/chloroform extraction and isopropanol precipitation. 50 ng aliquots of this DNA were used as template for PCR with bait-specific primers containing Illumina adapter termini (primer sequences and optimal PCR conditions available on request). PCR reactions were pooled, primers removed by washing with 1.8× AMPure XP beads, then quantified on a Bioanalyzer (Agilent) before sequencing with a HiSeq 4000 (Illumina).

#### Pre-Processing 4C Data for 4See

All bait sequence (including and downstream of the primer sequence, up to but not including the GATC *Dpn*II site) are trimmed by the demultiplexing Sabre tool (https://github.com/najoshi/sabre), allowing two mismatches, before mapping to the mm9 genome with Bowtie (Langmead et al., 2009) (**Figure 1B**). Interaction calling was done with peakC (Geeven et al., 2018) with different window sizes as specified by the parameter wSize. For previously published 4C results (Narendra et al., 2015), fastq files were downloaded from the Gene Expression Omnibus and processed just like the other data sets.

#### Analysis and Plotting of 4C Data Sets by Other Methods

Three 4C data sets (two replicates from ES cells; one replicate from *in vitro* differentiation of ES cells towards NPCs) were analyzed and plotted by 4Cseqpipe (van de Werken et al., 2012), r3Cseq (Thongjuea et al., 2013), fourSig (Williams et al., 2014), FourCSeq (Klein et al., 2015), 4C-ker (Raviram et al., 2016), and peakC (Geeven et al., 2018), using the default or recommended parameters given within the documentation accompanying the tools.

### 4See

#### System Requirements

4See is a GUI written in R (version > = 3.2), with the following packages (and their dependencies) additionally required, found on Bioconductor and/or CRAN: tcltk2, tkrplot, limma, caTools, rtracklayer. All scripts and test data are available under the terms of the GNU General Public License, version 3, on Github: https://github.com/TomSexton00/4See. The GUI is launched by sourcing the main script, 4See.r, from within an R environment. From then on, all manipulation is performed from a windows interface, and does not require use of command prompts. A full user manual in pdf format is also found with the scripts on Github, and is provided as **Supplementary Data**.

#### Input Format

4See deals with a simple text format, which we term the “cis” format, entailing a header with three tab-delimited fields (data set name, bait chromosome, bait coordinate) followed by a two-column table, denoting the coordinate of the mid-point of every restriction fragment found on the same chromosome as the bait, and its corresponding number of supporting sequence reads. The cis format is generated by a perl script, coord2frag.pl, provided with 4See, which maps the genomic coordinates of 4C sequencing results into their corresponding restriction fragments and then counts the number of reads for each fragment. The perl script accepts any non-headed text format for sequences, as long as a column for chromosome, coordinate, and strand can be specified. The restriction fragment information is provided by “frag” tables, headed four-column tables, giving a unique integer identifier, the chromosome, coordinate, and fragment length for each restriction fragment. These in turn are generated by a provided perl script, makefrags.pl, requiring a user input for the sequence of the primary restriction enzyme cutting site, and a folder containing the sequences in fasta format for each chromosome of the genome assembly used. The header of the cis file provides the required information on the bait location and 4C data set name, but is also used to ensure that only 4C data sets for the same bait (with identical bait chromosome and coordinate) are treated together.

#### Managing Conditions and Replicates

**Figure 3:**
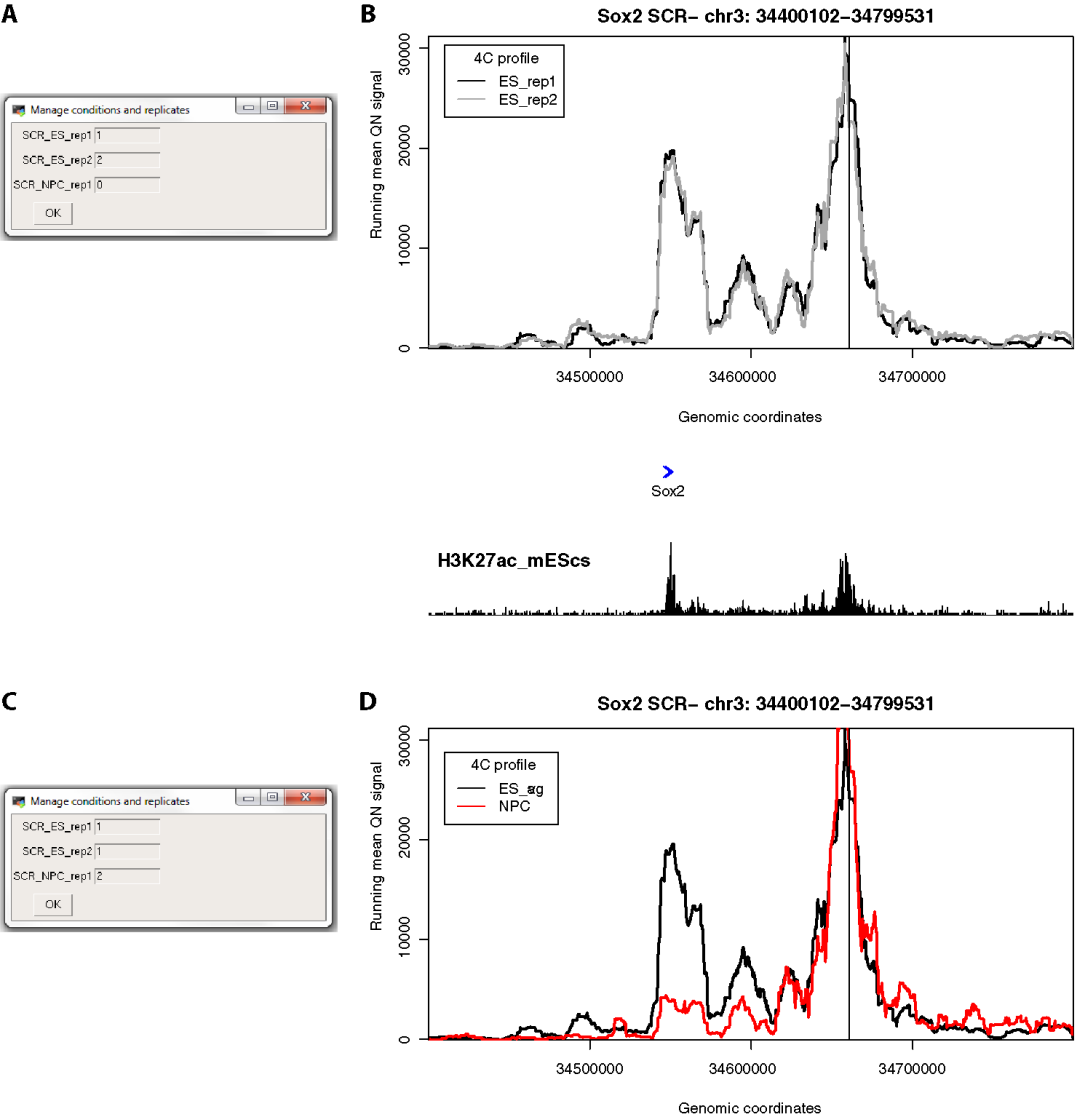
4See provides flexibility in handling multiple replicates and/or experimental conditions. **(A)** The 4See dialog box for conditions settings automatically opens when cis files are first loaded, in this instance two ES replicates and one NPC 4C data set for the *Sox2* SCR bait. The two ES replicates have been assigned different integers to be treated independently, and the NPC data set has been omitted by assigning it 0. **(B)** The resultant 4See plot from the conditions set in **(A)**, whereby the two ES 4C replicates are quantile normalized and plotted separately, one in black, the other gray. The plot has normalized 4C signal as the y-axis and genomic coordinate of the interacting fragment as the x-axis. The position of the SCR bait is denoted by a black vertical line, and gene position (blue arrows) and the ES H3K27ac ChIP-seq profile (black) is shown underneath the 4C plot. The profiles are highly consistent between replicates, with a strong interaction peak centered on the *Sox2* gene; note that both the gene and enhancer have a strong enrichment for H3K27ac. **(C)** As for **(A)**, but in this case the two ES replicates are given the same value to be averaged together, and the NPC data set is included as a different integer to the ES data sets. **(D)** As for **(B)**, but with the settings conditions of **(C)**, and the redundant gene and H3K27ac tracks omitted. The averaged ES 4C plot is given in black and the NPC 4C plot in red, showing a strong perturbation of the SCR-*Sox2* interaction on differentiation.

After loading one or more cis files, 4See opens a dialog box allowing the user to determine how to handle different conditions and replicates by assigning a value to each data set (**Figures 3A, C**). All 4C data sets assigned a non-zero integer are quantile normalized for fairer comparison across data sets (Ritchie et al., 2015). Data sets given the same value are averaged together before plotting; those assigned zero are omitted from normalization and plotting. Additional options allow the plotting color and data label to be specified by the user, and these can be re-run *via* the “Conditions” drop-down menu. Thus a user can rapidly compare different replicates side by side, or average them into one plot for comparison with different cell types or conditions, without needing to reload the data (**Figures 3B, D**).

#### Plot Settings

**Figure 4:**
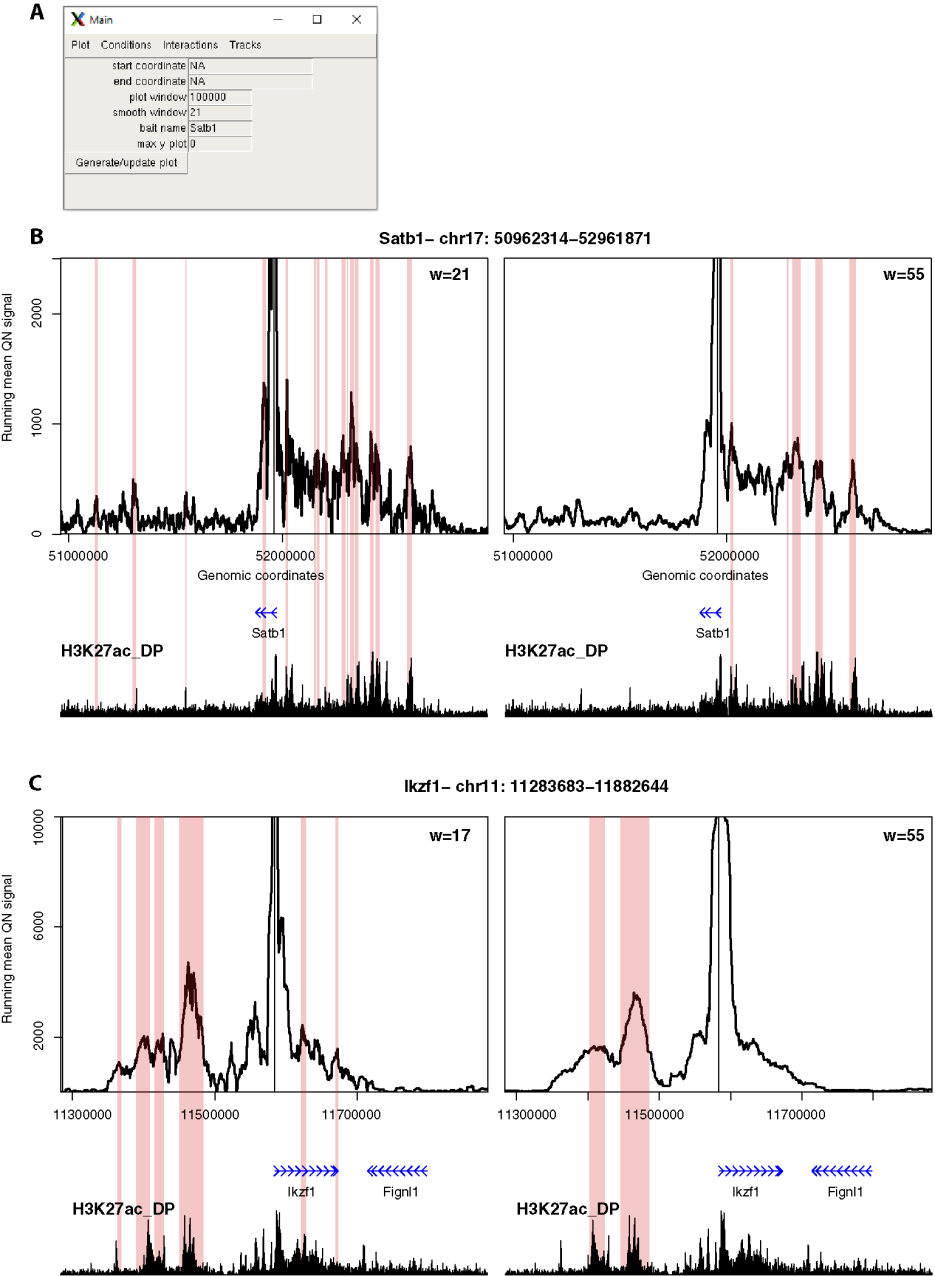
4See provides flexibility in running mean window sizes. **(A)** The main control panel of 4See, including options to set the x-axis (“start coordinate,” “end coordinate,” and “plot window”) and y-axis (“max y plot”) plot limits, to choose a bait name for the plot title (“bait name”), and to set the running mean window size (“smooth window”) in number of restriction fragments. **(B)** 4See plots for a 4C data set in DP thymocytes with the *Satb1* gene promoter as bait. Two instances of the same x- and y-axis limits are shown, with a running mean window of 21 (left) or 55 (right) fragments. The position of the *Satb1* bait is denoted by a black vertical line, and gene position (blue arrows) and the DP H3K27ac ChIP-seq profile (black) is shown underneath the 4C plots. Pink rectangles denote regions called as interacting by peakC for the equivalent window size as the plot. For the long-range interaction, the smaller window size appears to have more spurious called interactions, less evidently linked to H3K27ac peaks; the link is better seen with greater smoothing from a larger window size. **(C)** As for **(B)**, but with the *Ikzf1* gene promoter as bait. In this case, the smaller window size (17 fragments) seems to give better resolution of specific interactions with distinct putative enhancers, which are merged into one at larger window sizes.

4C profiles and the chromatin interactions they uncover differ with bait and experimental condition. In particular, the ease of distinguishing peaks of 4C signal above background depends on the distance of the interaction, since background signal of random chromosome collisions is much higher at shorter ranges (Dekker et al., 2002; Lieberman-Aiden et al., 2009). Other factors, such as whether the interaction is sharp with a single regulatory element, or is broadened across larger regions, such as “super-enhancers”, or the extent to which very short-range contacts dominate the plot and hide longer-range loops (which may be a consequence of the 4C digestion efficiency and/or relative compaction of the assessed locus), mean that features of chromatin topology are often overlooked with one fixed plot setting. The control panel of 4See includes options for the user to alter the region plotted, up to ± 1.5 Mb of the bait position, and to set a maximum plotted value on the y-axis (4C signal), to better visualize certain aspects of the 4C profile (**Figure 4A**). However a major confounding factor in visualizing 4C data is the need to smooth the plots, since “spikes” from spurious PCR duplicates make them appear very noisy at single restriction fragment resolution. Most analytical approaches counter this by taking running means (or medians) of sliding windows, but the results can be heavily influenced by the choice of window size. Reflecting this challenge, some 4C analytical tools adopt a “domainogram” approach, whereby averages are taken over many sliding windows of many different sizes, and the results are pooled together in a heat map (de Wit et al., 2008; van de Werken et al., 2012; see also **Figure 2A**), although the visual interpretation of these results is often challenging. To aid user choice in setting appropriate parameters for their particular 4C profile, 4See allows the window size (in numbers of restriction fragments) to be altered, and the appropriate running mean is calculated on the quantile normalized (and averaged, if replicates are pooled) data before plotting. In this manner, different aspects of chromosome topology can be readily explored (**Figures 4B, C**).

#### Annotations

**Figure 5:**
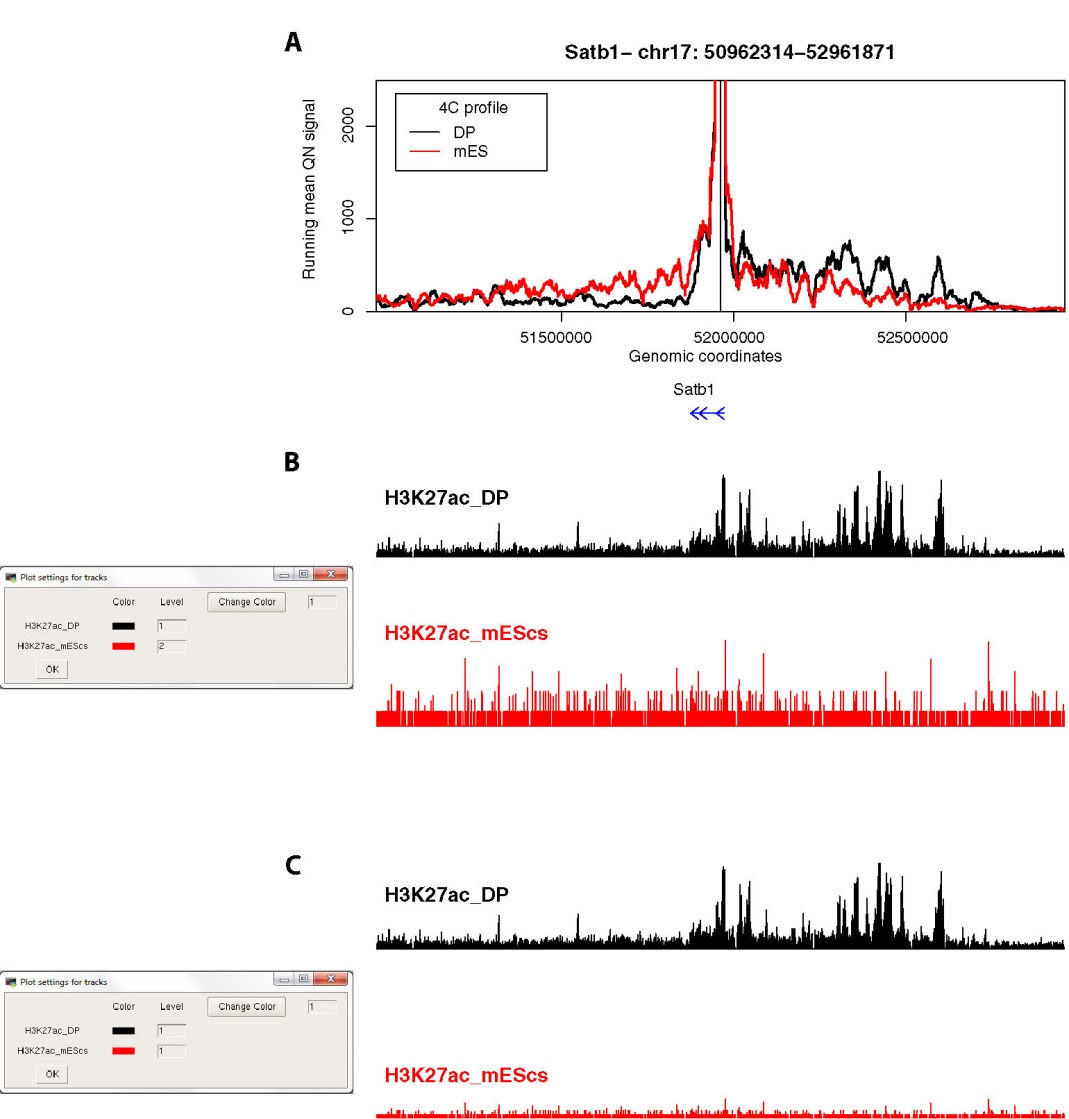
4See provides flexibility in handling epigenomic track scales. **(A)** The same 4C profile as **Figure 4B** (55-fragment window size; black) is plotted alongside the 4C profile for ES cells, where the locus is silent, and thymocyte enhancer interactions are not evident. **(B)** The 4See dialog box for managing epigenomic tracks defines how the different tracks are scaled. In this case, the ChIP-seq tracks for H3K27ac in ES and DP cells are treated independently, so the autoscaling of the ES track creates some spurious peaks from noise above background. **(C)** As for **(B)**, but this time the two H3K27ac tracks have been set the same integer, making the lower ES signal much more visually apparent in the plot.

To put the 4C profiles into a wider biological context, 4See supports the inclusion of three different types of annotations: genes, linear epigenomic profiles (termed “tracks”) and called interactions. Gene information is provided as a tab-delimited headed text file with the following fields: Name, Chr (with the prefix “chr”), Start, End, Strand (as “+” or “−”). When selected, the gene track is plotted in blue directly underneath the 4C profile. Only one gene track can be loaded at a time. Management of epigenomic profiles is more flexible. Any format supported by the *import* function of the rtracklayer package can be supported, but for running time efficiency we recommend loading bigWig files. As for the 4C profiles, the color and plotting level for each individual track can be altered by the user in an automatically loaded dialog box. As before, the plotting levels can be 0 (not plotted), or consecutive, positive integers. When tracks have the same level, their plots are auto-scaled to the maximum value of all of the included data sets within the plotted window. This feature allows fairer comparison for the same epigenetic mark across different conditions/tissue types (**Figure 5**). Technically, the numbers of tracks that can be loaded is only limited by system memory, although the plots become difficult to visually interpret after more than four tracks are loaded at a time.

To better highlight interactions called by existing peak-calling methods, or indeed to test how different methods and/or their parameters perform on specific 4C profiles, interactions (as bed files or similar, with headed “chr”, “start,” and “end” columns) can also be loaded, and these are represented as rectangles flanking the relevant region on the 4C profile (**Figures 4B, C**). A dialog box allows the user to alter the color of the annotation and to check whether or not it is plotted. The latter feature is useful since simultaneous plotting of more than one interactions set, which often overlap, can be difficult to visually interpret. Note that whereas these are labeled “Interactions” by 4See, any region described by a bed file can be highlighted in this manner. The user can thus use this setting to highlight any feature of interest, such as called differential interactions between two 4C data sets, or the presence of specific sequence motifs (e.g. CTCF) that may be expected to be enriched at interactions.

#### Exporting 4See Results

Once the user settings have been finalized, a pull-down menu option allows the plot to be saved in.eps format, where it can be further processed in preparation of a figure for publication or presentation. Alternatively, the data that are actually plotted in the current 4See window (one or more quantile-normalized 4C profiles with a running mean of a specified window size applied) can be exported as bedGraph files, ready for integration into other browsers, such as local instances of UCSC (Kent et al., 2002) or IGV (Robinson et al., 2011).

## Results

We demonstrate the usefulness of 4See on different original and previously published 4C data sets. First, we investigate the interaction between the mouse *Sox2* gene and an established cluster of enhancers (the “SCR”, or *Sox2* control region), which has been shown to be essential for *Sox2* expression in pluripotent cells (Zhou et al., 2014). Using bait primers at the SCR, we generated two biological replicates for ES cells and one after *in vitro* differentiation (“NPC”; **Figure 3**). As expected, we observed a strong interaction with the *Sox2* gene which is greatly reduced on differentiation. After loading the three data sets into the 4See browser, only changing the options within one dialog box is required to switch the view from plotting the two ES biological replicates side by side (omitting the differentiated data set) to confirm that they have consistent profiles, to comparing the averaged ES profile with the differentiated one.

Second, we explored different distance ranges of promoter-enhancer interactions at key developmental genes in mouse CD4^+^/CD8^+^ (double-positive, DP) thymocytes, namely the distal (~500 kb) enhancer cluster for *Satb1*, and the shorter-range (~50 kb) enhancer for *Ikzf1* (**Figure 4**). Comparing 4C plots at different running mean window sizes, it is apparent that different insights can be gained, and that no one window size is optimum for all profiles. For *Satb1*, shorter window sizes create what appear to be noisy profiles at the large genomic span assessed, and specific interactions are harder to discern. When the running mean window size is increased, the profile becomes smoother, and apparent peaks line up well with putative enhancers, as denoted by the presence of H3K27ac. Conversely, at the shorter distances assessed at the *Ikzf1* locus, a smaller window size allows interactions with specific enhancers to be resolved, whereas they merge into one large peak at larger window sizes. In support of this observation, we called interactions using the peakC algorithm (Geeven et al., 2018) at different window sizes, and found a good visual corroboration between discernible peaks and called interactions. 4See allows rapid re-plotting of 4C profiles with different window sizes, and also has the functionalities for adding the epigenomic profile and highlighting called interactions directly on the plot.

Third, we compared the same *Satb1* promoter-enhancer interaction between DP thymocytes, where the gene is highly expressed, and ES cells, where the gene is silent (**Figure 5**). As expected, the gene does not make any specific contacts with the thymocyte enhancer in ES cells. This locus is largely devoid of H3K27ac in ES cells, but a common problem with some browsers is that an automatic scaling creates some apparent peaks from noise on a small range of the y-axis (**Figure 5B**). 4See counters this by providing flexibility with how the epigenomic tracks are handled. By coercing the two tracks to the same scale, the difference between the two tissues is much more evident (**Figure 5C**).

**Figure 6:**
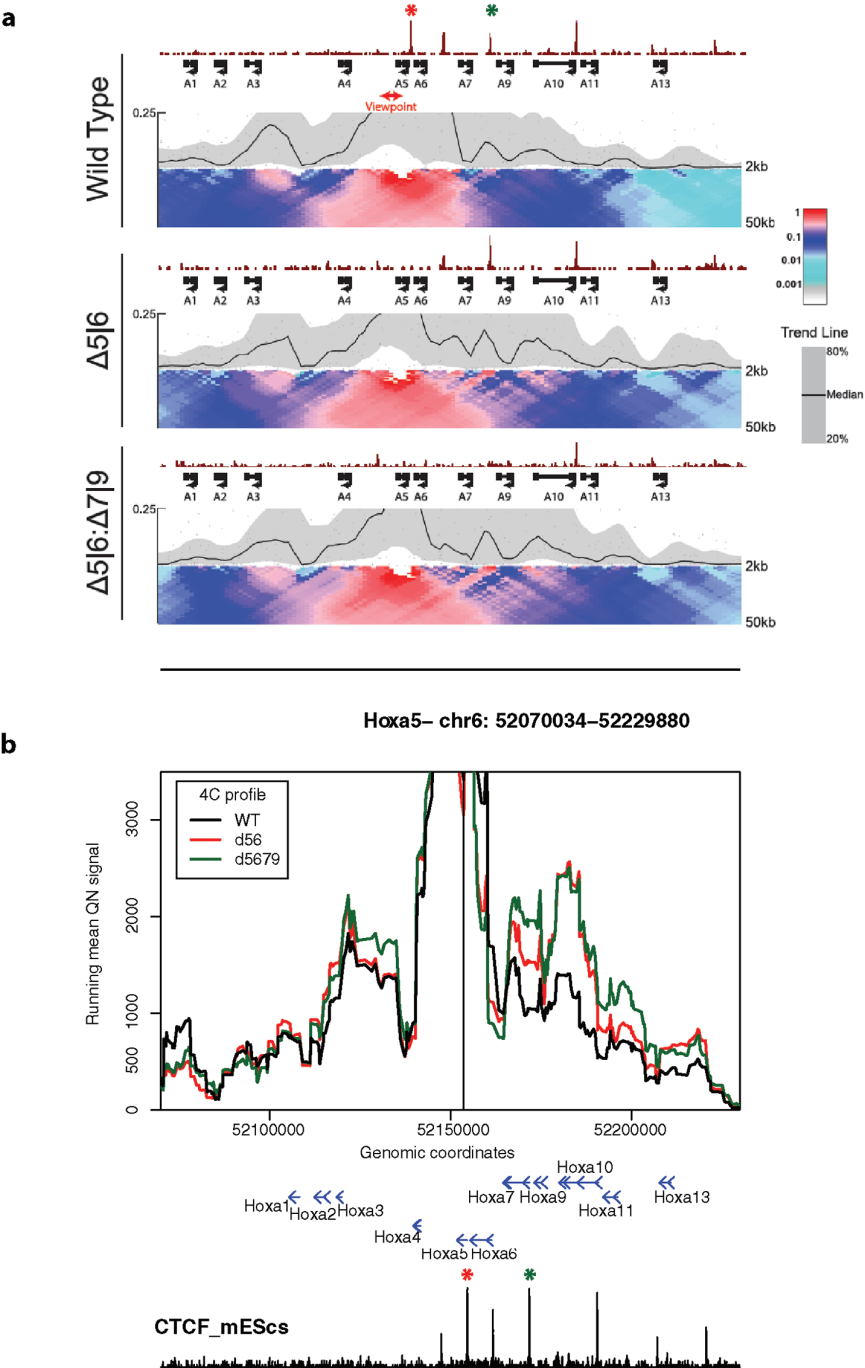
4See exploration can uncover previously overlooked features of chromatin topology. **(A)** Reproduced Supplemental Fig 7A from Narendra et al. (2015), used with permission. 4C profiles from the *Hoxa5* bait are shown as domainogram heat maps for wild-type ES cells (top), as well as lines that have had deletions of one CTCF site (middle; site of deletion denoted by red asterisk) or two (bottom; additional deletion site denoted by green asterisk). The CTCF ChIP-seq profile is shown above the 4C sets. No chromatin topology differences are apparent between these cell lines, and the original study concluded that spatial phenotypes only occurred on cell differentiation (Narendra et al., 2015). **(B)** The same data, processed and plotted using 4See. The position of the *Hoxa5* bait is denoted by a black vertical line, and gene position (blue arrows) and the ES CTCF ChIP-seq profile (black) is shown underneath the 4C plots. Red and green asterisks denote the positions of the single and double CTCF site deletions, as for **(A)**. In this plot, CTCF-dependent restriction of interactions between *Hoxa5* and more caudal regions (e.g. the gene body of *Hoxa10*) seems apparent.

Finally, we used 4See to re-analyze published 4C data, namely comparing profiles from the *Hoxa5* gene between wild-type ES cells and those where one or more key CTCF insulator sites have been deleted (Narendra et al., 2015). In this study, the authors reported that CTCF site loss caused topological defects during differentiation to neurons, with inappropriate spreading of H3K27me3. However, their analyses concluded that the topology of the *Hoxa* locus was largely unchanged in pluripotent cells (**Figure 6A**). Plotting the same data with 4See, it appears that ectopic looping interactions are formed between *Hoxa5* and more caudal regions of the locus (**Figure 6B**). Different loop calling algorithms with different parameter choices were inconsistent in calling these apparent interactions as “significant”, and only one biological replicate was available, so the importance of this observation is yet to be confirmed. In any case, the CTCF site deletion did not alter H3K27me3 patterning or *Hoxa* gene expression within undifferentiated ES cells (Narendra et al., 2015), so any potential topological changes do not appear to be borne out in other phenotypes. However, we wish to use this example to highlight how the use of a flexible browser like 4See facilitates exploration of the data, potentially identifying new features that “one size fits all” algorithms may overlook.

## Discussion

Using novel and previously published data sets for demonstration, we have shown the flexibility and utility of 4See in exploring 4C data. With limited processing of sequencing results, and one line of code in the R prompt, a user-friendly windows-based interface is available for a broader community to explore chromatin interaction profiles. As a consequence, we envisage that 4See will be of great use to the chromatin field. The input cis files are not very large (~ 4 MB for mouse or human), so the browser can be run on most desktop computers and laptops. The major systems limitation comes from the importing of epigenomic tracks (which can be >500 MB) with the rtracklayer package, which is the slowest step and may overload some standalone computers if too many tracks are imported at once. If the user is interested in only a specific set of baits, the system load can be reduced by restricting imports to chromosome-specific tracks. Due to the reliance of 4See plotting on quantile normalization, which is confounded by an excessive number of zeros or very small values, 4See is not an appropriate tool for visualizing very long-range (>1.5 Mb) or interchromosomal interactions; although their built-in graphical capabilities are more limited, the tools linked to algorithms such as fourSig should be used instead (Williams et al., 2014; Walter et al., 2019). It should also be noted that 4See does not replace the existing suite of interaction calling algorithms (Walter et al., 2019). Indeed, due to its capacity to incorporate these algorithms’ results into the plots, 4See should be viewed as a complementary tool for comprehensive 4C analysis, whereby the results of the algorithms can be readily visualized and compared to epigenomic tracks for validation and obtaining biological insight. Overall, 4See, in conjunction with other analytical tools, promises to facilitate chromatin interaction exploration, and will thus be of use to the epigenetics community.

## Data Availability Statement

Raw 4C data and the processed cis files first described here are available on GEO (GSE137417); previously published 4C results (Narendra et al., 2015) are available as data set GSE60240. ChIP-seq tracks were obtained from GEO for mouse ES (Shen et al., 2012) (CTCF and H3K27ac; GSE29218) and DP cells (Vanhille et al., 2015) (H3K27ac; GSE63732).

## Author Contributions

YBZ and TS designed and wrote the 4See code and accompanying scripts. AP and AM performed 4C experiments and preprocessing. TS wrote the manuscript, with continuous feedback from all authors.

## Funding

This study was supported by funds from the European Research Council (ERC) under the European Union’s Horizon 2020 research and innovation program (Starting Grant 678624-CHROMTOPOLOGY), the ATIP-Avenir program, and the grant ANR-10-LABX-0030-INRT, a French State fund managed by the Agence Nationale de la Recherche under the frame program Investissements d’Avenir ANR-10-IDEX-0002-02. YBZ is supported by funds from LabEX INRT, la Region Grand Est and the ERC. AP is supported by funds from LabEX INRT, the ERC, and the Ligue Nationale Contre le Cancer. AM is supported by funds from IDEX (University of Strasbourg) and the Institut National du Cancer. TS is supported by INSERM.

## Conflict of Interest

The authors declare that the research was conducted in the absence of any commercial or financial relationships that could be construed as a potential conflict of interest.
