# Supplementary material for: 4See: A Flexible Browser to Explore 4C Data
Source: Front Genet. 2020 Jan 21;10:1372. doi: 10.3389/fgene.2019.01372 (PMC6985583; doi:10.3389/fgene.2019.01372)
Supplement: Supplementary file 1 [file DataSheet_1.pdf]

Supplementary Data

4See: a flexible browser to explore 4C data – User Guide

System requirements and test data

4See is run on R, version >=3.2. These packages (and their dependencies) are additionally required, and can be found in Bioconductor or CRAN:

- tcltk2
- tkrplot
- limma
- caTools
- rtracklayer

If 4See is to be run on a remote server, X11 forwarding to the screen is also required for display of the graphical windows and plots.

For use of the *utils* scripts (see below), perl is required. The following files have been been included in the folder *testdata* for the user to trial the browser and accompanying scripts. Note that due to space limitations on github, any gzipped files will need to be unzipped before use.

- ES_SCR_rep1.cis, ES_SCR_rep2.cis and NPC_SCR.cis, three 4C datasets in the cis format (see below), which can be loaded directly into 4See. These have the *Sox2* SCR as bait, and represent two biological replicates from ES cells and one replicate from ES cells after *in vitro* differentiation to neural precursors.
- mm9_genes_chr3.txt, a truncated file containing the gene annotations for mouse chromosome 3 (see below).
- ES_SCR_interactions.bed, a bed file containing called interacting regions for the SCR in ES cells, as determined by peakC (Geeven et al., 2018).
- H3K27ac_ES_chr3.bw, a truncated bigWig file giving the H3K27ac ChIP-seq profile for chromosome 3 of mouse ES cells.

And for testing the *utils* scripts:

- mm9_DpnII_chr3.frags, a file containing the DpnII restriction fragment map for mouse, genome assembly mm9, restricted to chr3 for space reasons (see below).
- ES_SCR_rep2.map.gz, the output of sequence alignment of one of the 4C datasets (gzipped).
- A folder, *fasta*, containing the sequence of mouse chromosome 19, in fasta format (gzipped).

Input format

4See recognizes 4C data in a simplified, cis format. This entails a three item header, comprising the dataset name, then the chromosome and coordinate of the bait position. This is followed by a two-column table, where the first column is the coordinate of the midpoint of the restriction fragment and the second column is the number of 4C sequence reads mapping to that fragment. This is done for all restriction fragments on the same chromosome as the bait. For example, the beginning of ES_SCR_rep1.cis looks like:

```
SCR_ES_rep1      chr3  34660500
3001946    0
3002468    0
3002657    0
3003084    0
3003699    0
3004074    0
3004831    0
```

The script from *utils*, coord2frag.pl, can generate cis files from any text file containing the results of sequence alignment of a 4C dataset, provided it is non-headed, tab-delimited and contains columns with the following information: chromosome, coordinate, strand.

Usage perl coord2frag.pl [FRAGMAP] [READS_FILE] [CHR_COLUMN] [COORD_COLUMN] [STRAND_COLUMN] [CIS_OUTPUT_FILE] [BAIT_CHROMOSOME] [BAIT_COORDINATE] [DATASET_NAME] [READ_LENGTH].

The script reads in the READS_FILE file, extracting the chromosome, coordinate and strand information from the mapped reads, as defined by their column numbers (specified in CHR_COLUMN, COORDINATE_COLUMN and STRAND_COLUMN), then converts the coordinate to a particular restriction fragment, provided in the lookup table within FRAGMAP (see below). Rare reads that do not map to a particular restriction fragment are filtered out, as are restriction fragments that contain more than 2% of the total reads (most likely a result of undigested 4C material, and/or extreme PCR duplicate artefacts). The reads mapping to each fragment on the bait chromosome are then output to the cis file.

Example (assuming all the files and the perl script are in the same directory, which is the current directory):

```
> perl coord2frag.pl mm9_DpnII.frags ES_SCR_rep1.map 1 2 3 test.cis
chr3 34660500 ES_SCR_rep1 27
```

Output to the screen:

```
Reading frags file mm9_DpnII_chr3.frags…
391361 frags read in
Reading in ES_SCR_rep1.map…
1000000
2000000
3000000
4000000
5000000
```

```
6000000
7000000
8000000
Writing test.cis…
Ignored frag: chr3: 34660524 - 28.126981366792 %
0 discarded reads out of 8971080
```

And the file test.cis is created in the appropriate cis format. The FRAGMAP file comprises a headed tab-delimited table of the restriction fragments from a particular combination of genome and restriction enzyme. The column headers are: FRAG_ID (a unique integer ID for each fragment), CHROM, COORD, FRAG_LEN (the restriction fragment length). For example, the start of mm9_DpnII_chr3.frags looks like:

```
FRAG_ID CHROM   COORD   FRAG_LEN
3484194   chr3  3001455   982
3484195   chr3  3002437   63
3484196   chr3  3002500   314
3484197   chr3  3002814   541
3484198   chr3  3003355   689
3484199   chr3  3004044   61
3484200   chr3  3004105   1453
3484201   chr3  3005558   42
3484202   chr3  3005600   2737
3484203   chr3  3008337   83
```

New ones can be generated by the script from *utils*, makefrags.pl, provided the genome sequence is available in a dedicated directory containing one fasta file per chromosome.

Usage perl makefrags.pl [RESTRICTION_SITE] [FOLDER CONTAINING CHROMOSOME FASTA FILES] 30 [FRAGMAP_FILE]

The sequence for the smallest mouse chromosome, chr19, is found within the *fasta* folder to test the script:

```
> perl makefrags.pl GATC fasta 30 test_DpnII.frags
```

Output to the screen:

```
Extracting GATC
will read fasta/chr19.fa
done reading fasta/chr19.fa, got 61342436 bps
counted 143272 at chrom19
```

And the start of the file test_DpnII.frags looks like:

```
FRAG_ID CHROM   COORD   FRAG_LEN
1       chr19   3000604         39
2       chr19   3000643         908
3       chr19   3001551         770
```

```
4       chr19   3002321         370
5       chr19   3002691         172
6       chr19   3002863         384
```

Initializing the 4See browser

Load an R environment and navigate to the directory containing the 4See.r file, then load it:

```
> source("4See.r")
```

Two windows will then open, one where the profile will be output:

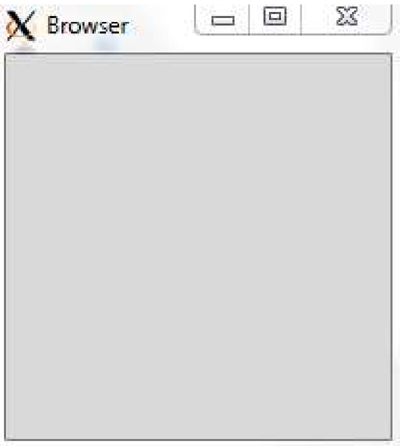

And a control panel, with various pull-down menus (explained below):

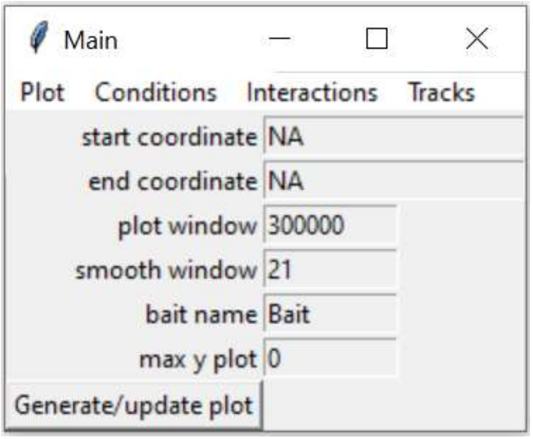

The four pull-down menus have the following options:

1. Plot

This menu has four commands:

i. Load 4C dataset(s). Clicking this opens a dialog box to browse and select one or more 4C dataset files to load.

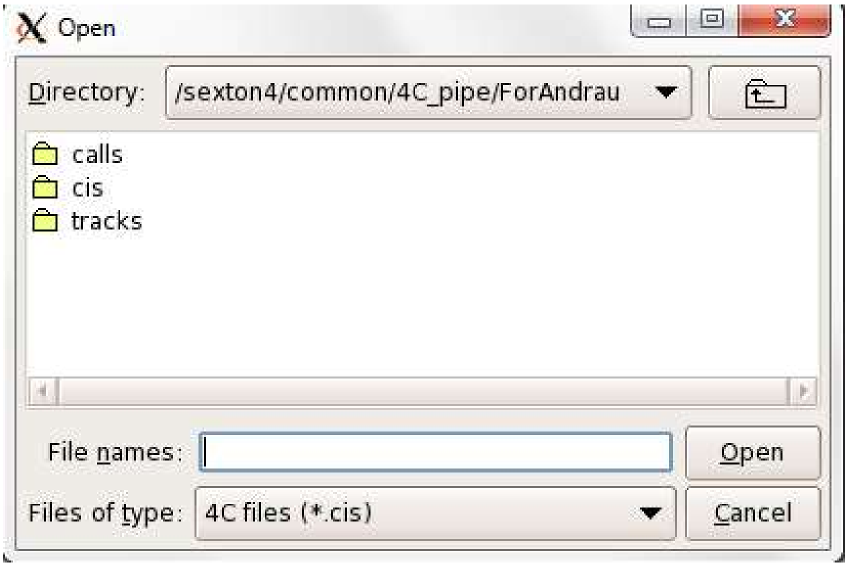

The *testdata* folder has three cis files that can be chosen. One or more cis files can be selected and input, although the bait chromosome and coordinate in the files' headers must be the same, otherwise an error message is given. Once loaded, a new dialog box opens for the user to select the *Conditions* settings to use (see also Conditions drop-down menu options, below):

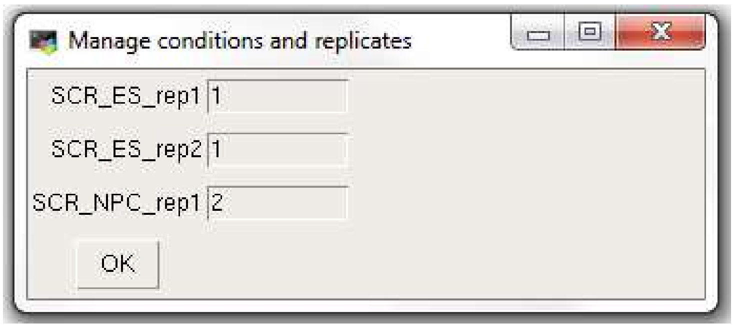

The user then determines how the different 4C datasets will be treated together by assigning each one either a 0 (omitted from analysis and plot) or a positive integer. Datasets given the same integer are pooled and averaged to one plot (see also **Fig 3**). In the above example, the NPC dataset is compared side-by-side with the average of the two ES datasets.

ii. Save Image. When a profile screenshot is ready, clicking this option opens up a dialog box for the profile to be saved as an electronic postscript (.eps) file.
iii. Save Bedgraph(s). When a profile screenshot is ready, clicking this option outputs the actual plotted values as bedgraph files to the current directory. One bedgraph file is made for each plot, with the name determined by the user-set name for each plot (see Conditions drop-down menu).
iv. Quit. Closes the graphical windows.

2. Conditions

This menu has two commands:

i. Set Conditions. Opens the same dialog box as when the datasets were first loaded. This allows plotting conditions to be changed (e.g. whether plotting two replicates side-by-side, or just plotting the average of them) without re-loading the source data.
ii. Plot Conditions. Opens a dialog box allowing the legend name and/or plot color to be changed:

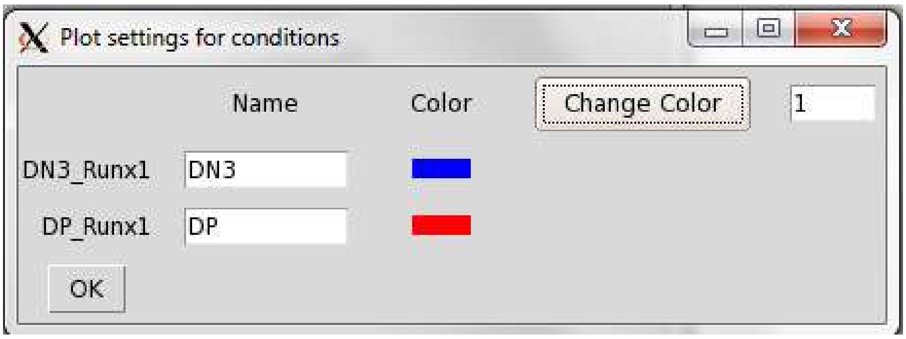

The legend name can be altered directly in the text box. If bedgraphs are saved (see above), the files will have these given names with the suffix .bedgraph. To change plot colors, select the number of the row to alter in the box adjacent to the “Change Color” button, then press the button. This will open a dialog box providing many different ways to choose the plot color, including palettes and swatches (the exact nature of the dialog box varies depending on the operating system used).

3. Interactions

For dealing with highlighting particular regions on the 4C plots. Note that while this feature is designed for highlighting called 4C interactions, any feature that can be defined by chromosome and coordinates can be used. This menu has two commands:

i. Load Interactions. Opens a dialog box for the user to select one or more bed files of interactions to load for highlighting on the plot (see **Fig 4**). These must be headed files containing the columns “chr”, “start” and “end”. The *testdata* folder has one interaction file that can be used in tests.
ii. Manage Interaction Plots. Similarly to Conditions, this opens a dialog box for the user to change either the legend name or color of the highlight, or to check whether or not the interaction set is annotated on the plot.

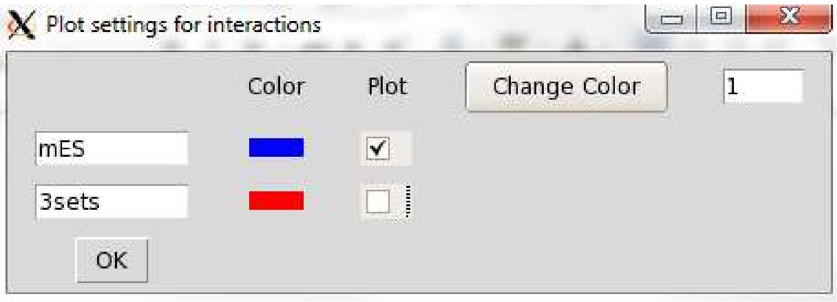

4. Tracks

This menu has three commands:

i. Load Genes. Opens a dialog box to load a gene annotation file. In the *testdata* folder, one can load mm9_genes_chr3.txt. The required format is a headed, tab-delimited text file, with the following columns: Name, Chr, Start, End, Strand. The Chr is given in the format chr1, chr2, …, chrX, and the strand must be "+" or "-". Only one genes file can be loaded. These are then plotted as blue arrows underneath the 4C plot.
ii. Load Tracks. Opens a dialog box to load one or more epigenomic tracks. In the *testdata* folder, there is a truncated ES H3K27ac ChIP-seq track that can be loaded. Any format that can be recognized by the R rtracklayer *import* function should work, but bigWig format is recommended. Once the data are loaded, a dialog box is opened, which is identical to the one loaded by the following command.
iii. Manage Tracks.

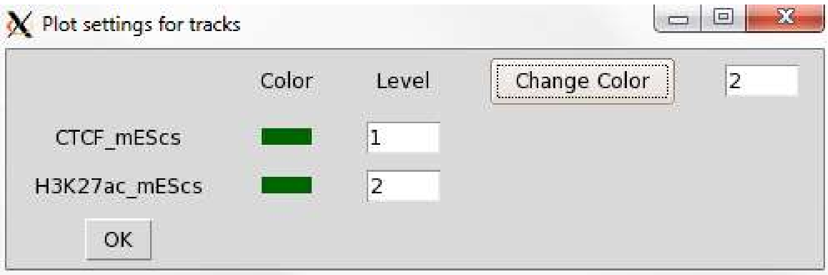

The color and plotting level for each individual track can be altered by the user. As before, the plotting levels can be 0 (not plotted), or consecutive, positive integers. When tracks have the same level, their plots are auto-scaled to the maximum value of all of the included datasets. This feature allows fairer comparison for the same epigenetic mark across different conditions/tissue types (see **Fig 5**).

Control Panel

Once all the data are loaded and conditions have been set, the control panel has options to choose:

- start and end coordinate. Defines the exact chromosomal coordinates within which the profile is plotted.
- plot window. As an alternative, the user can specify that the plot be a specific distance up- and downstream of the bait coordinate. If start and end coordinates have been set (i.e. are not "NA"), they override the plot window setting.
- smooth window. The number of restriction fragments to use when computing the running mean scores (which are what are actually plotted). Larger values give smoother-looking plots, but can cause some apparent interactions to be omitted (see **Fig 4**).
- bait name. This just alters the text that appears at the header of the plot.
- max y plot. The maximum y-value (running mean read count) to be plotted. When set to 0, this defaults to the maximum value within the plot. Since many 4C plots have extremely high read counts at regions very close to the bait, it is sometimes desirable to truncate the plot to make the distal interactions more evident.

Once everything has been loaded, click “Generate/update plot” to generate the plot. If multiple plot levels are included, they are quantile normalized for fairer comparison across datasets. To find the best plot for each dataset (or multi-dataset combination), the user will likely need to explore different smooth window and y max conditions, and possibly the plot coordinates.
